# Training and transfer effects of working memory training in male abstinent long-term heroin users

**DOI:** 10.1016/j.abrep.2020.100310

**Published:** 2020-11-05

**Authors:** Xin Zhao, Lei Wang, Joseph H.R. Maes

**Affiliations:** a Key Laboratory of Behavioral and Mental Health of Gansu Province, Northwest Normal University, Lanzhou 730070, China; b School of Psychology, Northwest Normal University, Lanzhou 730070, China; c Donders Institute for Brain, Cognition and Behaviour, Centre for Cognition, Radboud University, P.O. Box 9104, Nijmegen 6500 HE, the Netherlands

**Keywords:** Heroin addiction, Working memory training, Transfer effect

## Abstract

**Objective:**

Heroin abuse is associated with cognitive deficits. These might play a role in relapse after abstinence, which could be reduced by cognitive trainings. A previous study assessed effects of working memory (WM) training on executive functioning (EF) in heroin addicts undergoing methadone treatment, potentially limiting training effects. The present study assessed WM training effects in abstinent heroin addicts currently no longer receiving pharmacological treatment.

**Method:**

Inpatients were randomly assigned to a WM training or active control condition and performed EF tasks before and after training.

**Results:**

Trained participants improved their performance on the trained task. Using the control group as reference, they showed short-lived beneficial near- but no far-transfer effects to non-trained cognitive tasks. Participants with a strong baseline WM showed stronger training but smaller transfer effects than participants with a weak baseline WM.

**Conclusion:**

The combined results suggest limited cognitive transfer effects of WM training in heroin addicts irrespective of current methadone treatment status. They also suggest individual differences in training and transfer benefits dependent on baseline EF.

## Highlights

• Abstinent heroin abusers received working memory (WM) training or control treatment.
• Trained patients showed gain in performance on trained and transfer WM task.
• There was no evidence of far transfer effects.
• Individual differences in baseline WM capacity predicted training and transfer effects.
• Results match those of previous training studies with other (non-)addicted samples.

## 1 Introduction

Heroin is a widely abused drug across the world (Degenhardt et al., 2014), including China (Chen et al., 2019, Deng et al., 2011, Hao et al., 2002), and is associated with serious health and economic costs for both the individual and the society at large (Jiang, Lee, Lee, & Pickard, 2017). One of the individual’s health costs concerns brain damage and corresponding decrements in cognitive abilities. Previous studies found evidence for a direct link between heroin use and structural and functional brain changes and associated cognitive functions (Barash and Kofke, 2018, Fareed et al., 2017), and the longer the drug is used, the worse the user’s cognitive performance gets (Yuan et al., 2009, Yuan et al., 2010).

One important cognitive function that is affected is working memory (WM; Baldacchino, Balfour, Passetti, Humphris, & Matthews, 2012). WM capacity is associated with advanced cognitive abilities that enable the individual to deal with the demands of a complex world (Baddeley, 2010). WM capacity is closely related to executive control and selective attention, which enable the maintenance and processing of information despite distraction and potential interference (Engle, 2002, Gazzaley and Nobre, 2012, McCabe et al., 2010). WM is linked to prefrontal cortex-mediated top-down control of more basic, lower-level processes (Lara & Wallis, 2015), including spontaneous behavioral and emotional responses (Langner, Leiberg, Hoffstaedter, & Eickhoff, 2018). The compromised executive control fits the fact that heroin abuse results in decreased prefrontal cortex volume, thickness, and connectivity (Li et al., 2014, Mwansisya et al., 2016, Qi et al., 2011).

### 1.1 Improving executive functioning in addiction

Previous studies suggest that brain and cognitive impairments resulting from drug abuse in general, including heroin abuse, may play an important role in relapse of drug use after a period of abstinence (Li et al., 2016, Mizoguchi and Yamada, 2019, Zilverstand et al., 2018). Together with the premise of reversibility or recovery of the brain impairments and neurocognitive functions (plasticity; Lewis, 2017, Schulte et al., 2014), this suggestion has motivated research directed at enhancing the success of addiction treatments by improving cognitive abilities. This research includes studies using a training protocol targeting multiple aspects of executive functioning, or specifically WM capacity (e.g., see Bickel, Yi, Landes, Hill, & Baxter, 2011, and Brooks et al., 2017 for methamphetamine use, Houben, Wiers, & Jansen, 2011, and Khemiri, Brynte, Stunkel, Klingberg, & Jayaram-Lindström, 2019, for alcohol abuse, Loughead et al., 2016, for tobacco dependence, and Sweeney et al., 2018, for cannabis abuse). These studies found improved performance on the trained task(s) or (very) closely related tasks, but obtained mixed results in terms of performance benefits for less closely related cognitive tasks (so-called far transfer effects; Brooks et al., Khemiri et al., and Sweeney et al.: no or ambiguous transfer effects, and Bickel et al., and Loughead et al.: limited transfer effects). Mixed results were also obtained in these studies with respect to clinical outcomes (if measured; i.e., Loughead: no effects on smoking-related outcomes; Khemiri et al., and Sweeney et al.: effect on some but not all relevant clinical measures; Brooks et al.: self-reported beneficial effects on clinically relevant aspects; Houben et al.: reduced alcohol consumption).

### 1.2 WM training in opioid addiction

To our knowledge, only one study examined the effect of WM training in opioid-dependent individuals (Rass et al., 2015). The WM training was associated with a reduction of drug use and the trained individuals improved their performance on the trained tasks and on WM tasks closely related to the trained tasks (nearest transfer). However, there were no significant training-induced benefits (or at least not more than observed for control individuals performing a non-adaptive version of the training tasks) for cognitive tasks farther removed from the trained tasks, such as those assessing WM in another way (near transfer), or episodic memory, general intelligence, or delay discounting (far transfer). The lack of near- and far-transfer effects prompts the question as to the precise mechanism driving the positive training effects on drug use. Even more important for the purpose of the present study, this study concerned methadone maintenance patients, which may have reduced training effects because methadone itself has been shown to negatively affect brain function and structure, and associated cognitive functions (Li et al., 2016, Verdejo et al., 2005).

### 1.3 The present study

The aim of the present study was to assess training, transfer, and follow-up (maintenance) effects of WM training in a group of heroin-dependent patients that had been abstinent for at least 5 months and that currently were not undergoing any pharmacological treatment. Training effects were evaluated using a contact control group as reference. From a theoretical perspective, given the hypothesized methadone-induced (further) impairment of cognitive functioning, it could be expected that the WM training in the current sample results in stronger training and transfer effects as observed in Rass et al. (2015).

## 2 Methods

### 2.1 Participants

The participants were fifty abstinent heroin-dependent inpatients from Gansu Drug Rehabilitation Center (Gansu province, China). As part of the standard treatment provided by the center, all participants had received a three-month methadone maintenance treatment. However, for each of the included patients, this treatment had been finished 5–7 months before the start of the present study. Hence, at the time of the study, they no longer received any pharmacological treatment and none of the selected patients expressed a wish to resume such treatment. Their present treatment included non-pharmacological measures, such as physical exercise and psycho-social interventions. None of the selected patients received any other prescribed medication (e.g., against psychosis, depression, anxiety, or ADHD) that could affect cognitive performance. As part of the center’s admission regulations, the patients were not allowed to use alcohol, cigarettes, or any other addictive substances during their stay in the rehabilitation center. The participants were randomly assigned to either a training or control group (*n* = 25 for each group). A power analysis using G*Power 3.1.9.4. (Faul, Erdfelder, Lang, & Buchner, 2007) revealed that the required sample size for detecting a critical Group × Session interaction effect (see Data analysis below) with a power of 0.80, given an effect size of Cohen’s f = 0.25, *α* = 0.05, 2 groups, 2 measurements, and a correlation between measurements of 0.5, is 34 participants. The participants’ mean age, years of heroin use, and months of abstinence for each group are depicted in Table 1.

**Table 1:** Groups’ characteristics.

	CTRL	TRAIN	p*
N	25	25	
Age	46.9 (5.1)	45.1 (3.7)	0.15
Mean years of heroin use	21.7 (3.6)	20.0 (3.9)	0.11
Mean months of abstinence	6.0 (0.7)	5.9 (0.7)	0.44

Note: Values in parenthesis are standard deviations. Abstinence also included methadone that was previously provided in the framework of a methadone maintenance treatment. CTRL = control group; TRAIN = trained group. *p-value associated with *t*-test examining between-group difference.

**Fig. 1:**
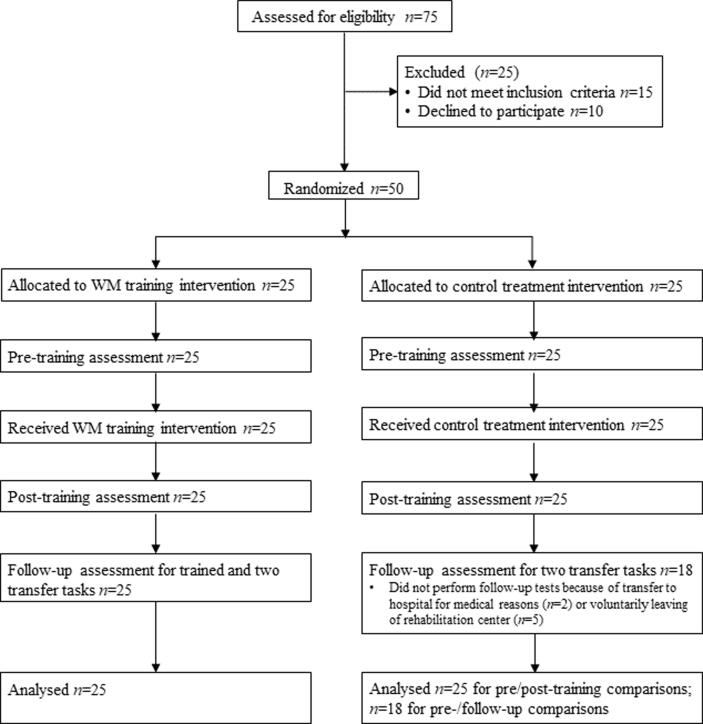
CONSORT flow diagram. WM = working memory training.

Seven participants from the control group did not perform the follow-up tests (see below). The two groups did not significantly differ on any of the three participant characteristics, as assessed with t-tests. All participants were literate and fluent in Chinese, had normal or corrected-to-normal vision, and had no physical or mental disorders (as confirmed by clinical staff and screening questionnaires) potentially affecting performance on the tasks. None of the participants had participated in a similar study before. All participants had received a detailed description of the procedures and provided written informed consent. This study was approved by the ethics committee of ***** and all procedures were in accordance with the approved guidelines. A CONSORT flow diagram is shown in Fig. 1.

### 2.2 Measures

The various cognitive tasks are only briefly described below; see Appendix A for more details.

#### 2.2.1 Training task

We adopted an adaptive visuospatial *n*-back task for WM training (e.g., see Studer et al., 2009). This task primarily demands WM monitoring and updating, one of the major components of executive functioning (Miyake et al., 2000), and is commonly used in WM training programs. On each trial, participants had to remember the spatial location of a stimulus on the computer screen and had to indicate whether the location of the present stimulus matched the position of the stimulus *n* trials back by pressing a key. The value of *n* changed based on performance accuracy. *N* was increased if the accuracy was > 90%, decreased if it was < 70%, and not changed in the other cases. The initial *n* level was 2. The dependent variable was the mean achieved *n*-back level on each training session.

#### 2.2.2 Pre- and post-training assessment tasks

##### 2.2.2.1 Flanker task

This task was adapted from the classic flanker task (Eriksen & Eriksen, 1974), and used to measure interference control. On each trial, drawings of five fish were presented in a horizontal row. Each fish could have one of two orientations. On congruent trials, all fish were oriented to the left or the right. On incongruent trials, the middle fish had a different orientation than the flanking fish. The participant had to respond to the orientation of the middle fish as fast and accurately as possible by pressing corresponding keys. The dependent measures were the mean response time (RT) on incongruent and congruent trials. A difference score was computed by subtracting the mean RT on congruent trials from the mean RT on incongruent trials. A higher score represents worse interference control.

##### 2.2.2.2 Go/no-go (GNG) task

An adapted version of the classic GNG task (see Donders, 1969) was used to assess response inhibition. On each trial, either the letter X or Y was presented. During the first half of the task, the participant had to respond as quickly as possible to each letter X but to refrain from responding to each Y. The (non-)response requirement was reversed in the second half (respond to each Y but not to X). The dependent measures were the percentage hits (correct response to go stimuli) and false alarms (incorrect response to no-go stimuli). We computed a hits minus false alarms difference score, with a higher value representing better response inhibition. The mean RT on go trials was used as index of general vigilance or decision-making speed.

##### 2.2.2.3 Color-word Stroop task

A color-word Stroop task (hereafter: Stroop task; MacLeod, 1991) was used as second measure of interference control. On each trial, either a string of four number signs or a Chinese character representing the color red or green, was shown. Each stimulus was printed in red or green. Participants had to indicate the stimulus’ print color as quickly and accurately as possible by pressing corresponding keys. On congruent trials, the color of the character matched its meaning; on incongruent trials the color and meaning did not match. A colored symbol string was presented on neutral trials. An interference score was computed by subtracting the mean RT on congruent trials from the mean RT on incongruent trials, with a higher score representing less interference control capacity.

##### 2.2.2.4 Running memory tasks (RMTs)

These tasks, the RMT-1750 and RMT-750 tasks (e.g., see Zhao et al., 2019), were used to measure WM ability. On each trial of the RMT-1750 task, a series of single digits were consecutively presented on a computer screen. The length of the sequence varied between 5, 7, 9, or 11 digits. On each digit presentation, which lasted 1750 ms, the participant had to memorize the final three digits of the sequence that had been presented so far. The participant had to enter the last three digits using the keyboard after the last digit of the sequence had been presented. The RMT-750 task was identical to the RMT-1750 task except that the digits were shown for 750 ms. The dependent measure for each task was the percentage of the total to-be-remembered target digits that were correctly retrieved (i.e., correct digit put into the correct serial position).

##### 2.2.2.5 Switching task

A classic switching task (e.g., see Monsell, 2003) was used to assess task-switching (or ‘cognitive flexibility’) capacity. On each trial, a digit was presented and the participant had to either indicate, by pressing corresponding keys, whether the digit was smaller or >5 (Task A: magnitude judgment) or whether the digit was odd or even (Task B: parity judgment). The current task requirement was indicated by the digit’s color. There were blocks of single-task trials and blocks of mixed-task trials, in which the task of the current trial was either the same as that of the preceding trial (non-switch trials) or different (switch trials). Two main dependent variables from switching tasks are the switch cost, the difference in RT or accuracy between switch and non-switch trials, and the mixing cost, the difference in RT or accuracy between non-switch trials from mixed-task blocks and single-task trials. Analyses of the RT and accuracy data revealed evidence of a speed-accuracy tradeoff. Therefore, we used the rank-ordering binning procedure (Hughes, Linck, Bowles, Koeth, & Bunting, 2014; see also Draheim, Hicks, & Engle, 2016 and Appendix B for further details) to combine the RT and accuracy data into one measure for the switch cost and one for the mixing cost. The higher the value, the larger the switch or mixing cost.

### 2.3 Procedure

The study had a pre-training assessment, training, and post-training assessment design. All participants first completed the flanker, GNG, Stroop, RMT, and switching tasks on 1–2 days. The participants from the training group then performed 20 training sessions within 20–21 days. During this period, the control group participants performed an equal number of sessions during which they made sand paintings (e.g., Zhao, Chen, & Maes, 2018). After the last training session, all participants again performed the transfer tasks on one single day in a fixed order. The participants also performed the RMT-1750 and switching tasks 60–70 days after the post-training assessment session (follow-up measurement). These tasks were included in the follow-up assessment because of the beneficial training effect observed for these tasks (for the switching task: when looking at the RT data, see Appendix B). During the follow-up assessment, the participants from the training group were also again tested on the *n*-back task used during training, to assess maintenance of training gains. All experimental procedures were performed in a standardized laboratory setting.

### 2.4 Data analysis

Training progress was analyzed using repeated measures analysis of variance (ANOVA) with training session as factor. For each transfer task, we performed a Group × Session (pre- vs. post-training assessment, or pre- vs. follow-up assessment), or Group × Trial Type × Session ANOVA on the corresponding outcome measures. We were specifically interested in interactions involving the group factor which, if significant, were followed-up by simple main effect analyses. In these analyses we used the error terms based on the overall analysis. In addition, we used analyses of covariance (ANCOVAs) in which group differences in post-training performance were evaluated using the pre-training performance score as covariate. For the trained participants, we also computed a pre- to post-training, and first- to last training session change measure for the various tasks. Spearman correlations among these *change* scores were computed to evaluate whether training-induced changes on one variable were related to changes on the other variables. An alpha level of 0.05 was used as criterion for statistical significance in all analyses. Effect sizes were expressed as partial eta-squared (*η_p_*^2^).

## 3 Results

### 3.1 Training progress

**Fig. 2:**
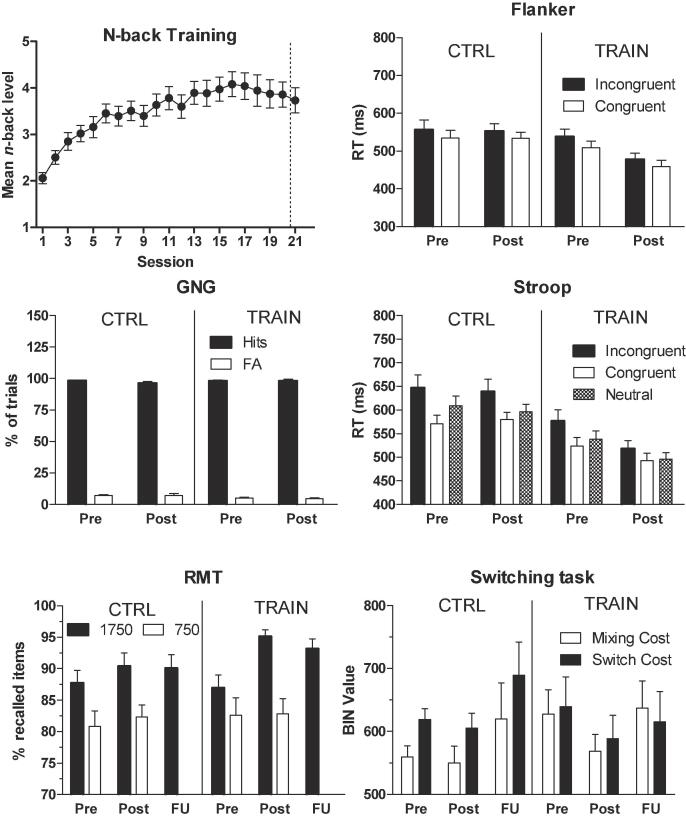
Top left panel: mean (+standard error of the mean, SEM) *n*-back level achieved by the trained patients on each training session and on the follow-up session. Remaining panels: mean (+SEM) score on the outcome measures of the flanker, go/no-go (GNG), Stroop, running memory (RMT), and the switching tasks, during the pre-treatment, post-treatment, and/or follow-up assessment sessions.

The top-left panel of Fig. 2 displays the training group’s mean *n*-back training task performance across the training sessions and the follow-up session.

There was a negatively accelerated performance increase and a maintained increased performance level at the follow-up measurement. ANOVA using the data of the 20 training sessions revealed a significant effect of session, *F*(19, 456) = 14.26, *p* < .001, *η_p_*^2^ = 0.37, which reflected a significant linear, *F*(1, 24) = 55.53, *p* < .001, *η_p_*^2^ = 0.70, and quadratic trend, *F*(1, 24) = 37.31, *p* < .001, *η_p_*^2^ = 0.61. Task performance during the follow-up session did not significantly differ from that on the last training session, *p* = .48.

### 3.2 Transfer task performance

The remaining panels of Fig. 2 show the groups’ performance on the outcome measures of the various transfer tasks, for the pre-training, post-training, and/or follow-up assessment sessions. For the flanker, Stroop, switching, GNG (hits/false alarms), and RMT-750 tasks, the ANOVAs did not reveal any significant critical interactions involving the group factor. However, these analyses confirmed the reliability of the flanker and Stroop tasks, revealing significantly faster responding on congruent than incongruent trials, *p*s < 0.001. Analysis of the RT on go trials did reveal a critical significant group × session interaction, *F*(1, 48) = 5.80, *p* = .02, *η_p_*^2^ = 0.11, which reflected a significant RT increase from the pre-training session (*M* = 391.3, *SD* = 33.9) to the post-training session (*M* = 407.2, *SD* = 36.6) for the control group, *F*(1, 24) = 4.85, *p* = .04, *η_p_*^2^ = 0.17, whereas the RT on the pre-training session (*M* = 386.9, *SD* = 39.4) did not differ from the RT on the post-training session (*M* = 378.3, *SD* = 37.1) for the trained participants, *p* = .24. Moreover, the trained participants responded significantly faster than the control participants on the post-training session, *F*(1, 48) = 7.73, *p* = .008, *η_p_*^2^ = 0.14, but not on the pre-training session, *p* = .68. The post-training between-group RT difference was also significant after controlling for pre-treatment RTs in the ANCOVA, *F*(1, 47) = 8.76, *p* = .005, *η_p_*^2^ = 0.16.

ANOVA on the pre- and post-training data of the RMT-1750 task revealed more accurate responding on the post- compared to pre-training session, *F*(1, 48) = 27.22, *p* < .001, *η_p_*^2^ = 0.36, which was further qualified by a significant group × session interaction, *F*(1, 48) = 6.95, *p* = .01, *η_p_*^2^ = 0.13. Simple main effect analyses revealed that the trained group performed better on the post- than pre-training session, *F*(1, 24) = 30.84, *p* < .001, *η_p_*^2^ = 0.56, whereas the control group did not display a significant performance difference on the two sessions, *p* = .08. The groups did not differ in pre-training performance, *p* = .77, whereas there was a trend towards better performance of the trained than control group on the post-training session, *F*(1, 48) = 3.08, *p* = .09, *η_p_*^2^ = 0.06. ANCOVA revealed significantly better post-training performance for the trained compared to control group, after controlling for the (slight) pre-training performance group difference, *F*(1, 47) = 8.84, *p* = .005, *η_p_*^2^ = 0.16. ANOVA using the pre- and follow-up performance data of the RMT-1750 task only revealed better performance on the follow-up compared to pre-treatment session, *F*(1, 41) = 18.38, *p* < .001, *η_p_*^2^ = 0.31, other *p*s > 0.08.

### 3.3 Bivariate correlations

Table 2 shows the correlations between the pre- to post-training or first to last training session *change* scores for the trained participants.

**Table 2:** Spearman correlations between the various measures.

	1	2	3	4	5	6	7
1. *N*-back	–						
2. Flanker	0.07	–					
3. GNG	0.23	−0.07	–				
4. Stroop	−0.17	−0.14	0.15	–			
5. RMT-750	−0.02	0.14	0.00	−0.15	–		
6. RMT-1750	−0.40*	0.26	−0.47*	0.05	0.31	–	
7. Switch-SC	0.06	−0.06	0.31	0.20	0.12	0.01	–
8. Switch-MC	−0.02	−0.08	0.13	0.07	−0.07	−0.02	0.77***

Note: Values indicate correlations between pre- to post-training or first to last training session (*N*-back task) *change* scores, based on the trained participants (*N* = 25). The change scores were computed such that high scores indicate better performance. The outcome measures of the tasks were: *N*-back task: mean achieved *n*-back level, flanker: incongruent/congruent trial RT difference score, GNG task: hits/false alarms difference score, Stroop task: incongruent/congruent RT difference score, RMT tasks: percentage recalled items, switching tasks: mixing and switching costs based on the binning procedure. SC = switch cost; MC = mixing cost. Values in bold are significant at *α* < 0.05; ***p < .001; *p < .05.

The change scores were computed such that higher scores represent a *larger* training gain, *less* interference during the flanker and Stroop task performance, *better* GNG response inhibition, *better* memory performance on the RMT tasks, and *less* switch and mixing costs. Larger pre- to post-training gains on the RMT-1750 task were associated with *smaller* training gains and less improvement on GNG task performance. Pre- to post-training changes in switch costs were positively associated with changes in mixing costs. None of the remaining correlations were significant.

## 4 Discussion

### 4.1 Summary of findings

Abstinent long-term heroin-dependent patients currently receiving non-pharmacological treatment participated in an adaptive visuospatial WM training program. They showed a steady improvement in performance on the trained task, which was still present 60–70 days after training. Using an active control group as comparison, the training also benefitted performance on the RMT-1750 task, although this benefit was no longer significant after 60–70 days. During the post-training GNG task, the non-trained patients performed slower than on the pre-training session, whereas such slowing down was not observed for the trained participants, even though the groups had equal performance accuracy levels. No transfer effects were seen for any of the other tasks. Exploratory correlation analyses revealed that pre- to post-training RMT-1750 performance changes were negatively correlated with training gain and improvement on the GNG task. In the next paragraphs, we compare the results of the present study with those reported by Rass et al. (2015).

### 4.2 Comparison of present findings with previous WM training study

As in Rass et al., the trained patients clearly showed performance improvements on the trained task across training sessions. In Rass et al., training progress across the 25 training sessions was expressed in terms of a learning slope, based on the average of the learning slopes for three WM tasks, including a visuospatial WM task as used in the present study. The reported slope was *M* = 0.04 (*SD* = 0.02). The average slope of the learning curves of the participants in the present study was *M* = 0.08 (*SD* = 0.06). A direct comparison of these slopes is complicated by the use of (partly) different training tasks and number of training sessions. However, a formal comparison, by expressing the regression weight difference as *z*-score (Paternoster, Brame, Mazerolle, & Piquero, 1998), revealed a significantly larger slope in the present study compared to the Rass et al. study, *z* = 3.33, *p* < .001. Arguably, training on a single task yields stronger training gains for that task compared to training progress on each individual task in a multiple-task training program.

In the present study, we found evidence of a short-lived near-transfer effect to RMT-1750 task performance. Performance on the digit-based RMT-1750 (but not the RMT-750 task) is held to reflect WM updating, as is the case with respect to the visuospatial WM task. However, the two tasks clearly have different formats, supporting the claim of a ‘near-’ instead of ‘nearest-’ transfer effect. This near transfer effect may be contrasted with the nearest-transfer effects found in the Rass et al. study, in which training only benefited performance on WM tasks that had the same format as the trained task. Despite this qualitative difference in transfer effects, the magnitude of the training-induced transfer benefits seemed to be similar in the two studies. That is, the effect sizes for the WM transfer tasks (pre- to post-training performance improvement) in the Rass et al. study were (based on the pooled *SD*s) Cohen’s *d* = 0.73 for the backward digit span task, and *d* = 0.52 based on the average of the three used versions of the visuospatial WM task (effect sizes based on information provided in Table 3 in Rass et al.). The effect size for the RMT-1750 task in the present study was *d* = 0.98. Comparing these effect sizes by applying Fisher’s *r* to *z* transformation, revealed no significant difference between the effect size for the RMT-1750 and backward digit span task, *z* = 0.40, *p* = .34, or between the RMT-1750 task and the visuospatial WM task, *z* = 0.74, *p* = .23. These results provide evidence for a somewhat more generalized training-induced transfer effect in the present compared to the previous study, although the effect sizes were similar. At present it is unclear whether this difference is due to differences in training gain or to the fact that, unlike the patients in the Rass et al. study, the present participants were no longer undergoing methadone treatment.

Further evidence that the RMT-1750 task performance benefit was training-induced comes from the correlation analyses. These revealed a significant association between WM training gain and RMT-1750 task performance improvement. However, this relation was negative rather than positive, which reflects the fact that participants with strong pre-training performance on the RMT-1750 displayed stronger training improvements than those with weaker initial RMT-1750 performance, while at the same time, there was less room for the former participants to further improve RMT-1750 task performance from pre- to post-training. This suggests a ceiling effect (see figure and analyses in Appendix C for illustration). These results suggest that WM training can especially improve performance on the trained task in individuals with relatively strong executive functions. This is in accordance with a magnification (“the rich will become richer”), as opposed to a compensation (“the poor will get richer”) account (Lövdén, Brehmer, Li, & Lindenberger, 2012). However, the results also suggest that such differential training gains may translate into a reversed effect in terms of transfer effects, depending on baseline performance differences on the transfer task, which may induce ceiling effects. The correlation analyses also suggest that performance on the RMT-1750 task requires processes partly involved in performing the GNG and switching tasks, as reflected in the negative correlation between gain on the RMT-1750 and GNG tasks. However, this correlation was present while there was no significant correlation between training gain on the *n*-back task and GNG task performance change.

Neither in the present study nor in the Rass et al. study was there any clear evidence of far transfer to other cognitive domains. In the present study, we found that the control participants performed more slowly on the go trials of the GNG task during the post- compared to pre-training assessment, whereas the trained participants continued to respond in the same speed. In combination with similar GNG accuracy rates in the two groups, the RT differences may suggest a training-induced enhanced response inhibition efficiency. However, the evidence is rather indirect and not supported by the correlation analyses (revealing no direct association between change in *n*-back and GNG task performance) and needs further validation in future studies. In any case, the lack of clear far-transfer effects in the two studies is in line with the general literature on WM training effects (Melby-Lervåg, Redick, & Hulme, 2016).

### 4.3 Limitations

Although the power analysis suggests that the present sample size is sufficient for detecting the critical interaction effects, the sample size was still relatively small in absolute terms. In this regard, the results are to be treated as preliminary. Unfortunately, we were not in the position to assess clinically relevant outcome measures, such as relapse rate. However, the study was primarily meant to examine the effects of WM training on cognitive measures in heroin-dependent patients that were currently free of any (substitute) drugs that could affect cognitive performances. Whether any training-induced changes also translate into improvements on clinical outcome measures remains a question for further research. The present study lacked a non-addicted control group, or a group of heroin addicts with less years of heroin use that would have allowed us to compare the magnitude of training and transfer effects of such groups with that of the present sample. Also, the comparison with heroin-dependent patients currently undergoing methadone maintenance treatment concerned a between- rather than within-study comparison. However, the results of the present study, revealing strong training but relatively limited transfer effects, agree with the general literature on the effects of cognitive training programs. Apparently, training effects are invariant across groups differing in heroin addiction status or current use or non-use of pharmacological substitutes.

## 5 Conclusions

To our knowledge, this is the second study examining effects of WM training in heroin-dependent individuals. Abstinent long-term heroin-dependent patients currently undergoing only non-pharmacological treatments participated in a WM training program. Relative to an active control group, these patients showed performance improvements on the trained task and short-lived beneficial transfer effects to a non-trained WM updating task. However, no transfer effects were found for tasks measuring interference control, response inhibition, and cognitive flexibility. The results of the present study, compared to those of a previous study on WM training in a sample of heroin-dependent patients receiving methadone, suggest somewhat broader transfer effects in the present sample. However, even the present transfer effects were limited, both in terms of breadth and duration. The present study also revealed some evidence of individual differences in both training- and transfer-task gains as a function of baseline WM capacity. These findings motivate followed-up research directed at the clinical value of training-induced transfer benefits and the possible role of individual differences in cognitive capacity therein.

**Contributors**

All authors contributed in a significant way to the manuscript and have read and approved the final manuscript. Dr. Zhao performed literature searches, designed the study, and prepared a first draft of the manuscript. Mr. Wang collected the data and performed initial data analyses. Dr. Maes was the author of the final draft, managed further literature searches, and performed the main analyses.

## Declaration of Competing Interest

The authors declare that they have no known competing financial interests or personal relationships that could have appeared to influence the work reported in this paper.

## References

[b0005] Baddeley A. (2010). Working memory. Current Biology.

[b0010] Baldacchino A., Balfour D.J.K., Passetti F., Humphris G., Matthews K. (2012). Neuropsychological consequences of chronic opioid use: A quantitative review and meta-analysis. Neuroscience & Biobehavioral Reviews.

[b0015] Barash J.A., Kofke W.A. (2018). Connecting the dots: An association between opioids and acute hippocampal injury. Neurocase.

[b0020] Bickel W.K., Yi R., Landes R.D., Hill P.F., Baxter C. (2011). Remember the future: Working memory training decreases delay discounting among stimulant addicts. Biological Psychiatry.

[b0030] Brooks S.J., Wiemerslage L., Burch K.H., Maiorana S.A., Cocolas E., Schiöth H.B., Kamaloodien K., Stein D.J. (2017). The impact of cognitive training in substance use disorder: The effect of working memory training on impulse control in methamphetamine users. Psychopharmacology (Berl).

[b0040] Chen K., Bian C., Song B., Gao G.e. (2019). Investigation and estimation of the prevalence of drug addicts in Xichang, China. Medicine.

[b0045] Degenhardt L., Charlson F., Mathers B., Hall W.D., Flaxman A.D., Johns N., Vos T. (2014). The global epidemiology and burden of opioid dependence: Results from the global burden of disease 2010 study: Epidemiology and burden of opioid dependence. Addiction.

[b0050] Deng Q., Tang Q., Schottenfeld R.S., Hao W., Chawarski M.C. (2011). Drug use in rural China: A preliminary investigation in Hunan Province: Drug use in rural China. Addiction.

[b0055] Donders F.C. (1969). On the speed of mental processes. Acta Psychologica.

[b0060] Draheim C., Hicks K.L., Engle R.W. (2016). Combining reaction time and accuracy: The relationship between working memory capacity and task switching as a case example. Perspectives on Psychological Science.

[b0065] Engle R.W. (2002). Working memory capacity as executive attention. Current Directions in Psychological Science.

[b0070] Eriksen B.A., Eriksen C.W. (1974). Effects of noise letters upon identification of a target letter in a non-search task. Perception & Psychophysics.

[b0075] Fareed A., Kim J., Ketchen B., Kwak W.J., Wang D., Shongo-Hiango H., Drexler K. (2017). Effect of heroin use on changes of brain functions as measured by functional magnetic resonance imaging, a systematic review. Journal of Addictive Diseases.

[b0080] Faul F., Erdfelder E., Lang A.-G., Buchner A. (2007). G*Power 3: A flexible statistical power analysis program for the social, behavioral, and biomedical sciences. Behavior Research Methods.

[b0085] Gazzaley A., Nobre A.C. (2012). Top-down modulation: Bridging selective attention and working memory. Trends in Cognitive Sciences.

[b0090] Hao W., Xiao S., Liu T., Young D., Chen S., Zhang D., Li C., Shi J., Chen G., Yang K. (2002). The second National Epidemiological Survey on illicit drug use at six high-prevalence areas in China: Prevalence rates and use patterns: Prevalence rates and patterns of illicit drug use in China. Addiction.

[b0095] Houben K., Wiers R.W., Jansen A. (2011). Getting a grip on drinking behavior: Training working memory to reduce alcohol abuse. Psychological Science.

[b0100] Hughes M.M., Linck J.A., Bowles A.R., Koeth J.T., Bunting M.F. (2014). Alternatives to switch-cost scoring in the task-switching paradigm: Their reliability and increased validity. Behavior Research Methods.

[b0105] Jiang R., Lee I., Lee T.A., Pickard A.S., Zhang H. (2017). The societal cost of heroin use disorder in the United States. PLoS ONE.

[b0110] Khemiri L., Brynte C., Stunkel A., Klingberg T., Jayaram-Lindström N. (2019). Working memory training in alcohol use disorder: A randomized controlled trial. Alcoholism: Clinical & Experimental Research.

[b0120] Langner R., Leiberg S., Hoffstaedter F., Eickhoff S.B. (2018). Towards a human self-regulation system: Common and distinct neural signatures of emotional and behavioural control. Neuroscience & Biobehavioral Reviews.

[b0125] Lara A.H., Wallis J.D. (2015). The role of prefrontal cortex in working memory: A mini review. Frontiers in Systems Neuroscience.

[b0130] Lewis M. (2017). Addiction and the brain: Development, not disease. Neuroethics.

[b0135] Li M., Tian J., Zhang R., Qiu Y., Wen X., Ma X., Wang J., Xu Y., Jiang G., Huang R. (2014). Abnormal cortical thickness in heroin-dependent individuals. NeuroImage.

[b0140] Li W., Li Q., Wang Y., Zhu J., Ye J., Yan X., Liu Y. (2016). Methadone-induced damage to white matter integrity in methadone maintenance patients: A longitudinal self-control DTI study. Scientific Reports.

[b0145] Li W., Zhu J., Li Q., Ye J., Chen J., Liu J., Li Z., Li Y., Yan X., Wang Y., Wang W. (2016). Brain white matter integrity in heroin addicts during methadone maintenance treatment is related to relapse propensity. Brain and Behavior.

[b0155] Loughead J., Falcone M., Wileyto E.P., Albelda B., Audrain-McGovern J., Cao W., Kurtz M.M., Gur R.C., Lerman C. (2016). Can brain games help smokers quit?: Results of a randomized clinical trial. Drug and Alcohol Dependence.

[b0160] Lövdén M., Brehmer Y., Li S.C., Lindenberger U. (2012). Training-induced compensation versus magnification of individual differences in memory performance. Frontiers in Human Neuroscience.

[b0170] MacLeod C.M. (1991). Half a century of research on the Stroop effect: An integrative review. Psychological Bulletin.

[b0165] McCabe D.P., Roediger H.L., McDaniel M.A., Balota D.A., Hambrick D.Z. (2010). The relationship between working memory capacity and executive functioning: Evidence for a common executive attention construct. Neuropsychology.

[b0175] Melby-Lervåg M., Redick T.S., Hulme C. (2016). Working memory training does not improve performance on measures of intelligence or other measures of “far transfer”: Evidence from a meta-analytic review. Perspectives on Psychological Science.

[b0180] Miyake A., Friedman N.P., Emerson M.J., Witzki A.H., Howerter A., Wager T.D. (2000). The unity and diversity of executive functions and their contributions to complex “frontal lobe” tasks: A latent variable analysis. Cognitive Psychology.

[b0185] Mizoguchi H., Yamada K. (2019). Methamphetamine use causes cognitive impairment and altered decision-making. Neurochemistry International.

[b0190] Monsell S. (2003). Task switching. Trends in Cognitive Sciences.

[b0195] Mwansisya T.E., Zhang H., Wang Z., Wu G., Hu A., Wang P., Liu Z. (2016). Major depressive disorder and heroin-dependent patients share decreased frontal gray matter volumes: A voxel-based morphometry study. International Journal of Emergency Mental Health and Human Resilience.

[b0200] Paternoster Raymond, Brame Robert, Mazerolle Paul, Piquero Alex (1998). Using the correct statistical test for the equality of regression coefficients. Criminology.

[b0205] Qi Y., Fu X., Qian R., Niu C., Wei X. (2011). Altered functional connectivity of prefrontal cortex in chronic heroin abusers. Neural Regeneration Research.

[b0210] Rass O., Schacht R.L., Buckheit K., Johnson M.W., Strain E.C., Mintzer M.Z. (2015). A randomized controlled trial of the effects of working memory training in methadone maintenance patients. Drug and Alcohol Dependence.

[b0225] Schulte M.H.J., Cousijn J., den Uyl T.E., Goudriaan A.E., van den Brink W., Veltman D.J., Schilt T., Wiers R.W. (2014). Recovery of neurocognitive functions following sustained abstinence after substance dependence and implications for treatment. Clinical Psychology Review.

[b0230] Studer, B. E., Jaeggi, S. M., Buschkuehl, M., Su, Y.-F., Jonides, J., & Perrig, W. J. (2009). Improving fluid intelligence – Single N-back is as effective as dual n-back. Poster presented at the 50th Annual Meeting of the Psychonomic Society. Boston, MA.

[b0235] Sweeney M.M., Rass O., DiClemente C., Schacht R.L., Vo H.T., Fishman M.J., Leoutsakos J.-M., Mintzer M.Z., Johnson M.W. (2018). Working memory training for adolescents with cannabis use disorders: A randomized controlled trial. Journal of Child & Adolescent Substance Abuse.

[b0240] Verdejo A., Toribio I., Orozco C., Puente K.L., Pérez-García M. (2005). Neuropsychological functioning in methadone maintenance patients versus abstinent heroin abusers. Drug and Alcohol Dependence.

[b0245] Yuan K., Qin W., Liu J., Guo Q., Dong M., Sun J., Tian J. (2010). Altered small-world brain functional networks and duration of heroin use in male abstinent heroin-dependent individuals. Neuroscience Letters.

[b0250] Yuan Y.i., Zhu Z., Shi J., Zou Z., Yuan F., Liu Y., Weng X. (2009). Gray matter density negatively correlates with duration of heroin use in young lifetime heroin-dependent individuals. Brain and Cognition.

[b0255] Zhao X., Chen L., Maes J.H.R. (2018). Training and transfer effects of response inhibition training in children and adults. Developmental Science.

[b0260] Zhao X., Fu J., Ma X., Maes J.H.R. (2019). Age differences in prospective memory: A further evaluation of the executive framework. Journal of Cognition and Development.

[b0265] Zilverstand A., Huang A.S., Alia-Klein N., Goldstein R.Z. (2018). Neuroimaging impaired response inhibition and salience attribution in human drug addiction: A systematic review. Neuron.

