# Supplementary material for: Training and transfer effects of working memory training in male abstinent long-term heroin users
Source: Addict Behav Rep. 2020 Nov 5;12:100310. doi: 10.1016/j.abrep.2020.100310 (PMC7752720; doi:10.1016/j.abrep.2020.100310)
Supplement: Supplementary data 1 [file mmc1.docx]

**Appendix A: Details of cognitive tasks**

**Visuospatial *n*-back Training task**

A 3 × 3 matrix was displayed on the screen. All cells were empty except for the middle cell that contained a fixation cross. On each trial, a blue square was randomly presented in one of the eight cells that surrounded the fixation cross. The stimulus was presented for 500 ms, with an inter-stimulus interval of 2500 ms. Upon presentation of the blue square, the participant had to indicate whether its position matched the position of the square *n* trials back. In case of a position match, the participant had to press the letter A on a keyboard. No response was required in case of a non-match. Task difficulty was reflected in the value of *n*: larger values represent a larger task difficulty. The *n* value was changed depending on the participant’s performance. If the accuracy was > 90%, *n* would be increased by 1. The value of *n* would be decreased by 1 if the accuracy was < 70%. Participants would continue with the same level in the other cases. All participants started with a 2-back task. Each session lasted about 25 minutes and consisted of 20 blocks, with 20+*n* trials in each block. The dependent variable was the mean achieved *n*-back level on each training session.

**Flanker task**

Each trial commenced with a 500-ms fixation cross, which was followed by a blank screen for a variable duration between 300 and 500 ms. Subsequently, a target stimulus was shown for 1500 ms or until the participant responded, whichever came first. The next trial started after a 1000-ms blank screen. Target stimuli consisted of five fish, which could occur in four different trial types. On congruent trials, all fish were either oriented to the left or to the right. On incongruent trials, the middle fish was oriented to the left of right, whereas the flanking fish were oriented to the other direction. The participant had to respond to the orientation of the middle fish as fast and accurately as possible by pressing the letter F for left and J for right. The main task was initiated after 16 practice trials which were repeated until the participant had reached an accuracy rate of > 85%. The main task consisted of four blocks of 32 trials. Sixteen congruent and 16 incongruent trials were randomly presented in each block. The dependent measure was the mean response time (RT) on incongruent and congruent trials. Trials with an incorrect response and a RT ≤ 200 ms were not included in the analysis. The outcome of the RT analyses reported in the main text are not compromised by a speed-accuracy tradeoff. One participant from the trained group had a relatively low accuracy rate (9.4%) on the pre-training test session and his data were not used in the RT analysis. The overall accuracy rate of the remaining participants was high (mean: 97.6%).

**GNG task**

Each trial began with a 100-ms fixation cross, after which the target stimulus, either the letter X or Y, was shown for 600 ms. The trial ended with a 1000-ms blank screen. The task started with 20 practice trials. The participant had to respond as fast as possible to each X by pressing the J key (go trials) but had to inhibit his response to each letter Y (no-go trials). This block of practice trials was repeated until the participant had achieved an accuracy level of ≥ 85%. The subsequent experimental phase comprised four blocks of 100 trials each. During Blocks 1 and 2, the participant had to respond to each X but not to Y. Trial Blocks 3 and 4 were preceded by one or more blocks of practice trials during which the participant had to respond to the letter Y but not to X. This practice block was repeated until the participant reached an accuracy level of ≥85%. During Blocks 3 and 4, the participant had to continue to respond to each Y and not to X. During each block, the letters X and Y were randomly presented and each go-letter (X or Y) was presented on 70% of the trials. The dependent measures from this test were the percentage hits (correct response to go stimuli) and the percentage false alarms (incorrect response to no-go stimuli). From these measures, we also computed a hits minus false alarm difference score. Finally, we also used the mean RT on go trials, excluding premature responses with RT ≤ 150 ms, as index of general vigilance or decision-making speed.

**Stroop task**

Each trial commenced with a black fixation cross that was shown for 500 ms. This was followed by a 1000-ms blank screen, after which the target stimulus was presented for 1500 ms. The trial ended with a blank screen, which was displayed for a variable duration between 600 and 1000 ms. Target stimuli consisted of a string of four number signs, or a Chinese character that either represented the color red or green. Each target stimulus was printed in either red or green. Participants had to indicate the print color of the target stimulus as quickly and accurately as possible, by pressing F for red and J for green. On congruent trials, the color of the character matched its meaning, for example, the character representing “red” was printed in red. Instead, on incongruent trials the color and meaning of the character were non-matching (e.g., the character representing “red” was printed in green). A colored symbol string was presented on neutral trials. Hence, there were six trial types: two congruent, two incongruent, and two neutral. First, a block of 18 practice trials was presented, which was repeated until reaching > 85% accuracy. Subsequently, three blocks of 36 trials each were presented. The six trial types were randomly but equally frequently presented, for a total of 36 incongruent, 36 congruent, and 36 neutral trials. The dependent measures were the mean RT on each of the three trial types. These RTs were based on trials with a correct response and a RT >200 ms. The overall mean percentage removed trials was 4.4% and the analyses based on the RTs reported below were not compromised by a speed-accuracy tradeoff (overall accuracy was 95.6%).

**RM-1750 and RM-750 tasks**

On each trial of the RMT-1750 task, a series of single digits, from 0−9, were consecutively presented on a computer screen. The length of the sequence varied between 5, 7, 9, or 11 digits. Each sequence lengths was presented an equal number of times in a random order. On each digit presentation, the participant had to remember the final three digits of the sequence that had been presented so far. The participant had to enter the last three digits using the keyboard after the last digit of the sequence had been presented. Each trial started with a 500-ms fixation cross, after which the digit was shown for 1750 ms. The digit-presentation ended with a blank screen, presented for a random time between 800 and 1200 ms. The pre-training task included three trial blocks. The first block consisted of eight practice trials. The second and third blocks each contained 12 trials. The post-training assessment session only included the two 12-trial blocks. The RMT-750 task was identical to the RMT-1750 task except that the digits were shown for 750 ms. Arguably, performance on the RM-1750 task reflects WM updating to a larger extent than is the case for the RM-750 task. A relatively long digit presentation time might encourage active monitoring and updating of the incoming digits, whereas short presentation times might hinder the use of this type of active processing and instead encourage recall from a more passive storage (e.g., see Bunting, Cowan, & Saults, 2006, and Zhao, Fu, Ma, & Maes, 2019, for supporting evidence, but see Broadway & Engle, 2010). The dependent measure for each task was the percentage of the total of 72 to-be-remembered target digits that were correctly reproduced, that is correct digit and put into the correct serial position.

**Switching task**

On each trial, one of the digits 1−9, except 5, was presented on the screen until the participant made a response. For each digit, the participant had to make either a magnitude judgement (Task A) or a parity judgement (Task B). The current task requirement was indicated by the digit’s color. A red color signaled the task to indicate whether the digit was larger or smaller than 5 (Task A), by pressing the A and L key, respectively. Instead, a blue digit indicated the task to judge whether the digit was odd (A key) or even (L key; Task B). The task commenced with Task A and Task B practice trials, which were repeated until reaching an accuracy rate of > 75%. Thereafter, the experimental trial blocks were initiated, consisting of 10 single-task blocks, and 10 mixed-task blocks. Each single-task block comprised 8 trials, whereas each mixed-task block consisted of 17 trials. During single-task blocks, either Task A or Task B was in effect, implicating a total of 80 single-task trials. In mixed-task blocks, pairs of Task A and Task B trials were alternated so that there were trials on which the current task was the same as the preceding task (non-switch trials), and trials on which the present task was different from the preceding task (switch trials), for a total of 80 non-switch and 80 switch trials. Two main dependent variables are derived from switching tasks, the switch and mixing costs. The switch cost refers to the difference in RT and/or accuracy between switch and non-switch trials. Relative to non-switch trials, switch trials demand additional cognitive resources, such as overcoming interference from the previous task or task set reconfiguration processes (Kiesel et al., 2010). Hence, switch trials are generally associated with longer RTs and/or more errors than non-switch trials. The mixing cost refers to the difference in RT and/or accuracy between non-switch trials from mixed-task blocks and single-task trials. Generally, the former trials are associated with longer RTs and/or more errors than the latter trials. The mixing cost is assumed to result from conflicts at the level of task sets or from an enhanced WM load during the non-switch trials (Rogers & Monsell, 1995; Rubin & Meiran, 2005). Analyses of the RT and accuracy data from the switch task in the present study revealed evidence of a speed-accuracy tradeoff. Specifically, when considering the RT data, the trained, but not control, group showed a training-induced reduction of the switch cost. However, this reduction was accompanied by an *enhanced* switch cost when looking at accuracy rates (see Appendix B). Therefore, we used the rank-ordering binning procedure developed by Hughes et al. (2014; see also Draheim et al., 2016, and Appendix B for further details) to combine the RT and accuracy data into one measure for the switch cost and one measure for the mixing cost.

**References**

Broadway, J. M., Engle, R. W. (2010). Validating running memory span: measurement of working memory capacity and links with fluid intelligence. *Behavior Research Methods*, *42*, 563−570. doi: 10.3758/BRM.42.2.563

Bunting, M., Cowan, N., & Saults, J. S. (2006). How does running memory span work? *Quarterly Journal of Experimental Psychology*, *59*, 1691−1700. doi: 10.1080/17470210600848402

Draheim, C., Hicks, K. L., & Engle, R. W. (2016). Combining reaction time and accuracy: the relationship between working memory capacity and task switching as a case example. *Perspectives on Psychological Science*, *11*, 133−155. [doi: 10.1177/1745691615596990](https://doi.org/10.1177%2F1745691615596990)

Hughes, M. M., Linck, J. A., Bowles, A. R., Koeth, J. T., & Bunting, M. F. (2014). Alternatives to switch-cost scoring in the task switching paradigm: their reliability and increased validity. *Behavior Research Methods*, *46*, 702−721. [doi](https://doi): 10.3758/s13428-013-0411-5

Rogers, D. R., & Monsell, S. (1995). Costs of predictable switch between simple cognitive tasks. *Journal of Experimental Psychology: General*, *124*, 207−231. doi: [10.1037/0096-3445.124.2.207](http://psycnet.apa.org/doi/10.1037/0096-3445.124.2.207)

Rubin, O., & Meiran, N. (2005). On the origins of the task mixing cost in the cuing task-switching paradigm. *Journal of Experimental Psychology: Learning, Memory, and Cognition*, *31*, 1477−1491. doi: 10.1037/0278-7393.31.6.1477

Zhao, X., Fu, J., Ma, X., & Maes, J. H. R. (2019). Age differences in prospective memory: a further evaluation of the executive framework. *Journal of Cognition and Development*, *20*, 680−701. doi: 10.1080/15248372.2019.1648268
