# Supplementary material for: Training and transfer effects of working memory training in male abstinent long-term heroin users
Source: Addict Behav Rep. 2020 Nov 5;12:100310. doi: 10.1016/j.abrep.2020.100310 (PMC7752720; doi:10.1016/j.abrep.2020.100310)
Supplement: Supplementary data 2 [file mmc2.docx]

**Appendix B**

*Figure 1*. Left: Mean RT (+SEM) on the three trial types during each of the three assessment periods, separately for the control (CTRL) and trained (TRAIN) group. Right: Mean percentage of correct responses on each of the trial types on each assessment session for the control and trained participants.

A Group × Session (pre- vs. post-treatment) ANOVA on the RT switch cost (SC: RT_switch trials_ - RT_non_-_switch trials_) revealed a significant interaction effect, *F*(1, 48) = 7.50, *p* = .009, *η_p_*² = .14, which reflected a smaller SC during the post-treatment assessment (*M* = 81.9, *SD* = 89.3) than the pre-treatment assessment (*M* = 227.8, *SD* = 179.2) for Group TRAIN, *F*(1, 24) = 21.25, *p* < .001, *η_p_*² = .47, but not for Group CTRL (*M* = 207.3, *SD* = 197.8, respectively, *M* = 244.2, *SD* = 201.2), *p* = .14. Also, the SC was smaller for Group TRAIN than Group CTRL during the post-treatment session, *F*(1, 48) = 8.35, *p* = .004, *η_p_*² = .15, but not the pre-treatment session, *p* = .76. The same pattern of results was found for the pre-treatment versus follow-up assessment comparison.

A Group × Session (pre- vs. post-treatment) ANOVA on the accuracy-based SC cost (Accuracy_non-switch trials_ - Accuracy_switch trials_) also revealed a significant interaction effect, *F*(1, 48) = 5.22, *p* = .03, *η_p_*² = .10, which reflected a trend towards a larger SC during the post-treatment session (*M* = 5.2, *SD* = 5.2) than the pre-treatment session (*M* = 2.9, *SD* = 5.4) for Group TRAIN, *F*(1, 24) = 3.78, *p* = .06, *η_p_*² = .14, but not Group CTRL (*M* = 1.4, *SD* = 4.5 respectively, *M* = 3.3, *SD* = 5.8), *p* = .19. Moreover, the accuracy SC was smaller for Group TRAIN than Group CTRL during the post-treatment session, *F*(1, 48) = 7.72, *p* = .008, *η_p_*² = .14, but not the pre-treatment session, *p* = .80. An identical analysis using the pre- and follow-up assessment accuracy data did not reveal any significant effects.

Group × Session ANOVAs using the RT- and accuracy-based mixing cost (MC: RT_non-switch_ - RT_single trials_, respectively, Accuracy_single trials_ – Accuracy_non-switch trials_) did not reveal any significant interaction effects, *p*s > .09.

The results of these analyses suggest a speed-accuracy tradeoff with respect to the SC, thereby motivating the use of a binning procedure in which RT and accuracy data are combined into one measure. Specifically, we used the rank-ordering binning procedure developed by Hughes et al. (2014; see also Draheim et al., 2016), to combine the RT and accuracy data into one measure for the switch cost and one measure for the mixing cost as dependent variables in the primary analyses. Briefly, a low switch cost bin score indicates that accurate switch trials are only moderately associated with longer RTs relative to the average non-switch trial, and relatively few errors on switch trials. Similarly, a low mixing cost bin score implies that accurate non-switch trials are not strongly related to longer RTs compared to the average single-trial RT and that there are not many errors on non-switch trials.

**References**

Draheim, C., Hicks, K. L., & Engle, R. W. (2016). Combining reaction time and accuracy: the relationship between working memory capacity and task switching as a case example. *Perspectives on Psychological Science*, *11*, 133−155. [doi: 10.1177/1745691615596990](https://doi.org/10.1177%2F1745691615596990)

Hughes, M. M., Linck, J. A., Bowles, A. R., Koeth, J. T., & Bunting, M. F. (2014). Alternatives to switch-cost scoring in the task switching paradigm: their reliability and increased validity. *Behavior Research Methods*, *46*, 702−721. [doi](https://doi): 10.3758/s13428-013-0411-5
