# Supplementary material for: Training and transfer effects of working memory training in male abstinent long-term heroin users
Source: Addict Behav Rep. 2020 Nov 5;12:100310. doi: 10.1016/j.abrep.2020.100310 (PMC7752720; doi:10.1016/j.abrep.2020.100310)
Supplement: Supplementary data 3 [file mmc3.docx]

**Appendix C**

*Figure 1.* Left panel: mean achieved *n*-back (±SEM) level during the training and the follow-up session for participants scoring either low or high on the pre-training RMT-1750 task. The two subgroups were created using a median split. Right panel: Mean percentage (+SEM) of recalled items during the pre-treatment, post-treatment, and follow-up assessment for trained participants scoring high (black bars) or low (white bars) on the pre-treatment session (based on median split).

The regression equation associated with the learning curve for the low and high RMT-1750 subgroups (left panel of Figure 1) were, respectively, y = .063 * X + 2.39, and y = .101 * X + 2.92. A regression analysis using subgroup (low vs. high RMT performers), session, and the subgroup * session interaction term as predictors of the mean *n*-back level revealed a significant effect for the interaction term, *t* = 2.15, *p* = .03, indicating a significantly larger slope for the high- compared to low-RMT task performance subgroup.

A Group × Session (pre- vs. post-training) ANOVA on the RMT-1750 data (right panel of Figure 1) revealed a significant interaction effect, *F*(1, 23) = 21.61, *p* <.001, *η_p_*² = .48, reflecting a significant pre- to post-training performance improvement for the low RMT-1750 performance group, *F*(1, 12) = 40.91, *p* <.001, *η_p_*² = .77, but no improvement for the high RMT-1750 performing group, *p* = .10. The same subgroup difference in performance gain applied to the pre-training versus follow-up assessment comparison.
